# The Therapeutic Engagement Questionnaire (TEQ): a service user-focused mental health nursing outcome metric

**DOI:** 10.1186/s12888-019-2326-x

**Published:** 2019-12-03

**Authors:** Mary Chambers, Sue McAndrew, Fiona Nolan, Ben Thomas, Paul Watts, Robert Grant, Xenya Kantaris

**Affiliations:** 1 Faculty of Health, Social Care and Education, Kingston University and St George’s, University of London, St George’s Campus, 6th Floor Hunter Wing, Cranmer Terrace, London, SW17 0RE UK; 2 School of Nursing, Midwifery, Social Work & Social Sciences, University of Salford, Salford, Greater Manchester, M6 6PU UK; 3 Camden and Islington NHS Foundation/University College London, Centre for Outcomes Research and Effectiveness, 1-19, Torrington Place, London, WC1E 7HB UK; 4 Department of Health, Strategy and External Relations Directorate, 79 Whitehall, London, SW1A 2NS UK; 5 Somerset Partnership NHS Foundation Trust, Community Mental Health Nursing, 2nd Floor Mallard Court, Express Park, Bristol Road, Bristol, TA4 4RN UK

**Keywords:** Therapeutic Engagement, Service Users, Registered Mental Health Nurses, Mental Health Nursing

## Abstract

**Background:**

Therapeutic engagement (TE) has been described as the crux of mental health nursing but despite its perceived importance, to date, there is no measurement tool that captures it. As a result, there is no way of determining the contribution of mental health nursing interaction to service user recovery, in acute inpatient mental health settings or the wider care quality agenda.

**Methods:**

To develop and validate a TE measurement tool in partnership with Service Users (SUs) and Registered Mental Health Nurses (RMHNs). The TEQ was developed in 3 stages: 1) item generation (and pre-testing), 2) item reduction using Principal Component Analysis (PCA), and 3) validation across Mental Health Trusts in England.

**Results:**

The final questionnaire has two versions, (SU and RMHN version), each scored within two contexts (1–1 SU-RMHN interactions and overall environment and atmosphere of the ward) and includes 20 items with two sub-scales (care interactions and care delivery). Psychometric evaluation of the TEQ shows high inter-scale correlations (0.66–0.95 SU; 0.57–0.90 RMHN), sound sub-scale internal consistency (> 0.95), concurrent validity (> 0.60) and adequate score variability for both versions of the TEQ. In summary, the TEQ behaves well as a measurement tool.

**Conclusions:**

The TEQ can determine the collaborative and empathic nature of RMHN-SU interactions, capture if SUs are treated with dignity and respect and recognise that the principles of the recovery approach are being respected. The TEQ can also provide robust monitoring of nursing activity, offer opportunity for transparency of activity, feed into healthcare organizations’ key performance indicators and provide reassurance about the nature and quality of nurses’ work.

## Background

Since the inception of Peplau’s (1952) [1] seminal work which emphasised the primacy of the nurse–patient relationship, therapeutic engagement (TE) has been considered the crux of psychiatric nursing [2]. Current evidence suggests that TE is beneficial, and is of significant clinical importance [3–5]. Indeed, service users (SUs) value positive attitudes, being listened to, and being able to trust those who provide care.

Given the perceived importance of therapeutic engagement in mental health nursing it is necessary to evaluate how such engagement contributes to SU recovery and the overall quality agenda of healthcare organisations [6, 7]. Such a metric has not yet been developed, consequently there is no way to measure the nature of ‘face-time’ and TE as part of the SU experience as perceived by SUs and registered mental health nurses (RMHNs).

An array of rating scales exist to measure therapeutic engagement with a variety of titles, for example the Working Alliance Inventory (WAI) [8], and the Helping Alliance Scale (HAS) [9]. Their tendency is either to measure TE within research [10], or measure the qualitative nature, of TE, making it difficult to quantify and assess the quality. The Scale To Assess the Therapeutic Relationship (STAR) [10], was developed to assess the relationship between multidisciplinary clinicians and SUs who experience mental illness; despite its merits it was not designed in partnership with SUs in acute inpatient mental healthcare settings [10, 11]. Whilst the co-production of tools in partnership with SUs is in its infancy, such involvement gives greater credibility to the final product.

From the tools identified, none of them assess the 1-1 SU-RMHN interactions in acute inpatient mental healthcare settings nor the overall environment and atmosphere of the ward.

The aim of this study was to develop such a tool using psychometric methodology, resulting in a short and simple tool that can quantify and recognise nursing engagement activity in the monitoring and enhancement of SU care and recovery.

## Methods

### Study design

#### Stage 1 of 3 – item generation

A previous article by Chambers et al. (2016) [12] provides an explicit description of the questionnaire, the two versions and the two contexts. In this article, stage 1 of the development and validation of TEQ entitled the item generation stage, which took place in co-production with SUs and RMHNs, is described in detail. In the item generation stage, the original 25-item (statement) questionnaire encompassing five themes (with a 4 point Likert scale response format) was generated based on the literature, feedback from a therapeutic engagement workshop (*n*=70) involving service users, clinical nurses and nurse academics, findings from the ‘Lived Experience of Detained Patients’ project (in-depth interviews with 19 detained service users) [13] and review of the tool (pre-testing) by both parties. Both parties liked the use of *‘me’ or ‘I’* in the statements and that the positively worded statements appeared to be sensitive to care interactions and delivery. Clarity of instructions, statement-stems and the 4-point Likert response options were also discussed and agreed. Clinical appropriateness of each statement was ensured by the RMHNs.

The TEQ has a SU and RMHN version and both are scored in relation to two contexts - SU-RMHN 1-1 interactions in acute inpatient mental healthcare settings and the environment and atmosphere of the ward^12.^

This article describes the next two stages of its development, item reduction and psychometric evaluation, of both versions of the tool.

#### Stage 2 of 3 – item reduction

In this stage, the questionnaire was administered to 86 SUs and 68 RMHNS from 4 Mental Health NHS Trusts across different regions of England. Standard item reduction techniques were then used to develop the sub-scales - care delivery and care interactions – in both versions of the TEQ.

### Procedure and participants

The newly revised TEQ which encompasses 20-items instead of the original 25 was administered face-to-face to 154 participants. Purposive sampling was adopted. Ward managers identified eligible SUs and RMHNs for the study who were then invited by the research team to participate in the study. Adult service users with the following eligibility criteria were invited to complete the SU version of the questionnaire within their care environment (with support from a person of their choice if needed who was not their named nurse): residing for more than one week within an adult acute inpatient mental healthcare setting, mental capacity to consent (as determined by the ward nursing staff and treating Psychiatrist using the four-point British Medical Association mental capacity test) and good command of the English language as the TEQ has been initially developed in English. Registered mental health nurses working in an acute inpatient mental health setting attached to the Mental Health NHS Trust participating in the study with a permanent work contract were invited to complete the revised nurse version of the questionnaire within their work environment. Data were collected within a 3-month period. The response rate was not calculated as the project was not able to access data on service users who had not consented to participate.

### Data Analysis

The data collected from the questionnaires underwent a Principal Components Analysis (PCA) with oblimin rotation. Principal Component Analysis (PCA) is adequate for the development of a measurement tool and is the most commonly used in exploratory factor analysis determining the underlying domains (factors and structural validity) of measurement tools [14]. Oblimin rotation is the standard method and allows the factor to be correlated which often provides sound factor structures. Factors were retained with eigenvalues over one, and items were chosen if the items loaded on the factor > 0.40 as per ‘rule of thumb’ [15].

## Results

Two factors were evident and were named care interactions and care delivery by our ‘expert’ group during the item generation stage. For the environment and atmosphere context of the SU version, 8 items fell under the sub-scale care delivery and 12 items in the care interactions sub-scale. For the 1-1 interactions context of the SU version of the TEQ, 9 items also fell under the care delivery factor and 11 under the care interactions factor.

For 1-1 interactions, two factors were retained - the first factor was very strong with 66% of variance. For the environment and atmosphere context of the ward, only one factor was retained (75% of variance). The loadings were not as clearly separated as we had previously observed [16]. Based on the statistical analysis at this stage of the development of the TEQ and SU feedback about all the items we saw no compelling reason to drop items prior to the validation stage.

The internal consistency for the sub-scales was examined using Cronbach’s α [17] estimates exceeded 0.80 which indicated good consistency [18]. The αs were very high (α = 0.98 for 1-1 interactions; 0.97 for environment and atmosphere context of the ward), which remained the same when each item was deleted one at a time from each sub-scale.

For both versions of the TEQ, missing items were low. Participants who had any missing data were deleted as per rationale for multivariate analysis. Seventy-two per cent, 79%, 81% and 92% respectively of respondents endorsed all 20 items (100% complete data). The number of respondents who failed to complete 3 or more items was very low (0.01%, 0.00%, 0.03%, 0.00% respectively). Therefore, scale scores could be computed for most of the respondents. Distribution for item response scales was symmetrical and not skewed and items within each scale had similar mean scores and standard deviations. All correlations between items and the total score were high (0.77-0.91). Scales scores spanned the entire scale range and were not notably skewed, mean scores were near the scale mid-point however floor and ceiling effects were moderately high (maximum 30.3%).

For the environment and atmosphere context of the RMHN version, 9 items fell under care delivery and 11 items in the care interactions sub-scales; these items were the same as the SU version. For the 1-1 interactions context of the RMHN version of the TEQ, 4 items also fell under the care delivery factor and 16 under the care interactions factor.

For 1-1 interactions, two factors were retained with eigenvalues over 1, and they explained 61% of the variance; the first factor was very strong with 66% of variance. For the environment and atmosphere context of the ward, two factors were retained (62% of variance). After rotation, both sets of responses were loaded similarly to the pattern observed for the service users.

The internal consistencies for the sub-scales were very high (α = 0.95 for 1-1 interactions; 0.96 for environment and atmosphere of the ward), which remained the same when each item was deleted one at a time from each sub-scale.

After item reduction, the 4-point Likert scale response format remained. It should also be known that at this stage both groups of participants were given a platform to state any problems with the wording and content of the statements, to produce any missing statements and/or topic areas to be included in the tool. There were no examples of this therefore the scale was not revised prior to analysis.

### Stage 3 of 3 – validation

In this stage, the psychometric properties of the TEQ i.e. data quality, scaling assumptions, acceptability, reliability and validity, were evaluated in a national sample (with wide geographical spread) of 628 SUs and 543 RMHNs. Tables 1 and 2 show the response data for each sub-scale of the service user version and nurse version of the TEQ.

**Table 1:** Data quality, scaling assumptions and acceptability of the service user version of the TEQ

Psychometric property	1:1 Care interactions	Care delivery	General Care interaction	Care delivery
Data quality (*n* = 628)				
No. of respondents missing data on 1 item only	42	42	52	34
No. of respondents missing data on 2 items only	21	13	26	9
No. of respondents missing data on 3 items or more	8	2	16	2
No. of respondents with complete data	454	496	510	575
Scaling assumptions				
Item Mean Score Range	1.68–1.99	1.65–2.03	1.72–2.18	1.75–1.98
Item SD Range	0.86–0.97	0.81–0.97	0.83–0.99	0.83–0.91
Item Skewness Range	0.66–1.22	0.65–1.51	0.44–1.14	0.62–1.09
Item correlation with Hypothesised Scale	0.84–0.91	0.82–0.90	0.77–0.88	0.78–0.87
Acceptability				
Total Possible Score Range	11–44	9–36	12–48	8–32
Total Observed Score Range	11–44	9–36	12–48	8–32
Mean Observed Score (SD)	20.4 (9.0)	15.6 (6.8)	23.4 (9.1)	14.7 (5.8)
Floor/Ceiling Effect^a^	30.3%	29.2%	16.5%	19.5%
Skewness	0.85	1.22	0.72	0.89

^a^% of participants with scores at the minimum or maximum

**Table 2:** Data quality, scaling assumptions and acceptability of the registered mental health nurse version of the TEQ

Psychometric property	1:1 Care interactions	Care delivery	General Care interaction	Care delivery
Data quality (*n* = 543)				
No. of respondents missing data on 1 item only	28	23	19	13
No. of respondents missing data on 2 items only	7	1	12	0
No. of respondents missing data on 3 items or more	5	22	2	0
No. of respondents with complete data	481	497	491	515
Scaling assumptions				
Item Mean Score Range	1.17–1.58	1.43–1.66	1.23–1.77	1.24–1.45
Item SD Range	0.45–0.68	0.57–0.73	0.51–0.76	0.53–0.62
Item Skewness Range	0.95–3.33	0.90–1.23	0.70–2.87	1.38–2.39
Item correlation with Hypothesised Scale Range	0.67–0.81	0.77–0.83	0.67–0.81	0.74–0.84
Acceptability				
Total Possible Score Range	17–68	3–12	12–48	8–32
Total Observed Score Range	17–68	3–12	12–48	8–32
Mean Observed Score (SD)	21.9 (6.5)	4.5 (1.5)	17.6 (5.5)	10.5 (3.5)
Floor/Ceiling Effect^a^	27.9%	34.2%	18.5%	36.7%
Skewness	3.31	1.17	2.28	3.30

^a^% of participants with scores at the minimum or maximum

### Procedure and participants

To assess the psychometric properties of the TEQ, the questionnaire was authenticated against two validated questionnaires that measure therapeutic alliance in community settings and research respectively, The Scale To Assess the Therapeutic Relationship (STAR) [10] and The Helping Alliance Scale (HAS) [18]. The STAR assesses therapeutic relationships in community psychiatry. The clinician version of the STAR has three subscales: Positive Collaboration, Emotional Difficulties, Positive Clinician Input. The patient version of the STAR also has three sub-scales: Positive Collaboration, Positive Clinician Input, Non-Supportive Clinician Input, and the HAS measures the strength of the patient-therapist therapeutic alliance.

The aim of the validation was to examine the evidence that the TEQ was a measure of therapeutic engagement for the populations mentioned. This authentication occurred with service users and RMHNs in 26 England Mental Health Trusts with wide geographical spread. Table 3 shows the participant characteristics which appeared to cover the diversity of service users and nurses. Eligible services users and RMHNs (as per the item reduction stage) from all 26 Trusts were invited to participate in this stage of the study by completing the three questionnaires either in their care or work environment.

**Table 3:** Characteristics of the participants at the validation stage

Variable	Service users	Nurses
n	628	543
Gender^a^		
Female	50	35
Male	33	8
Not stated	17	57
Ethnicity		
White British	28	40
Black or Black mixed	8	25
Asian or Asian mixed	7	5
Not stated	57	30
Age		
20–30	–	38
31–40	–	19
41–50	–	13
51–60	–	5
61–70	–	1
Not stated	–	24
Education		
Higher degree		7
University degree		48
University diploma		4
Other		5
Not stated		36
Grade		
Band 5		38
Band 6		13
Band 7		4
Band 8		1
Not stated		44

^a^All values from here onwards are in %

In total, 628 SUs and 543 RMHNs completed the appropriate version of the TEQ across the participating Trusts. The number of SUs and RMHNs who completed the whole HAS was 392 and 401 respectively; the number of SUs and RMHNs who completed the whole STAR was 445 and 453 respectively.

### Data analysis and Results

To help determine the TEQ’s adequacy, convergent validity was determined by examining the sub-scale correlations between the TEQ sub-scales in each version of the TEQ using Pearson’s product-moment correlation coefficient. We predicted that the sub-scales in each version of the TEQ and type of questioning of the TEQ would correlate highly (>0.70). Correlations ranged from 0.66-0.95. Strong correlations were also found in the RMHN version of the TEQ with the exception of the care delivery sub-scale in each of the contexts of the TEQ i.e. 1:1 and environment and atmosphere of the ward (0.57). Tables 4 and 5 show these correlations.

**Table 4:** Convergent validity of the service user version of the TEQ

	One to one		General	
	Care interactions	Delivery of care	Care interactions	Delivery of care
One to one				
Care interactions	–	–	–	–
Delivery of care	0.92 (0.87–0.95)	–	–	–
General				
Care interactions	0.84 (0.78–0.88)	0.77 (0.71–0.83)	–	–
Delivery of care	0.74 (0.66–0.80)	0.77 (0.71–0.82)	0.87 (0.82–0.91)	–

(95% bootstrap confidence intervals)

**Table 5:** Convergent validity of the registered mental health nurse version of the TEQ

	One to one		General	
	Care interactions	Delivery of care	Care interactions	Delivery of care
One to one				
Care interactions	–	–	–	–
Delivery of care	0.75 (0.69–0.89)	–	–	–
General				
Care interactions	0.82 (0.69–0.89)	0.75 (0.62–0.84)	–	–
Delivery of care	0.80 (0.64–0.90)	0.57 (0.41–0.68)	0.85 (0.79–0.89)	–

(95% bootstrap confidence intervals)

It should be known that limited numbers of SUs and RMHNs missed responding to 3 items or more.

Concurrent validity was determined by examining the sub-scale correlations between the TEQ sub-scales with the STAR and HAS. The analyses showed significant, moderate correlations (>0.60) in the 2 versions of the TEQ in both contexts. Given that the HAS and STAR were designed with other populations in mind, the direction, magnitude and pattern of the correlations are generally consistent.

## Discussion

The aim of this study was to develop a TE measurement tool that combines the service user perspective with a rigorous scientific approach. The TEQ includes 20 items with two sub-scales - care interactions and care delivery. The questionnaire is easy to administer, has versions for SUs and RMHNs and has satisfactory psychometric properties. The inter-scale correlations are high (0.66-0.95 SU; 0.57-0.90 RMHN) and the TEQ exhibits sound sub-scale internal consistency (>0.95). The authentication shows acceptable concurrent validity and is supported by significant, moderate correlations with the other measures used for authentication. The majority of the relationships between the sub-scales and authentication measures were expected however there are apparent weaker correlations between the care delivery sub-scales in both the contexts of the RMHN version of the TEQ. Reasoning could be that the nurses make a distinction between types of interaction within this sub-scale.

The questionnaire’s psychometric properties show that in general the TEQ behaves well as an assessment scale. Indeed, the TEQ has the capacity and necessary psychometric properties to measure and quantify TE in adult acute in-patient psychiatric settings from the perspectives of both SUs and RMHNs.

### Scoring and interpretation of the TEQ

For clinical purposes the TEQ should be completed at SU discharge. The SU and their primary/named nurse (with whom they should have had recovery-focused interactions however brief) will independently complete their respective version of the TEQ so that responses can be matched when reported. There is no scoring system attached to the TEQ given the number of items in each sub-scale and context for each version. Individual items and/or the groups of items in the sub-scales should be reviewed. The higher the score, the better the engagement. A holistic viewpoint is the most informative way to understand SU and RMHN scores. Although simplistic, this method ensures that service users' thoughts, feelings, beliefs, and attitudes are supported by nursing staff.

### Limitations

The TEQ was developed and validated in accordance with psychometric theory [19] and developed within the NHS in partnership with SUs, RMHNs and clinical nurse academics, therefore it is highly relevant and useful to clinical practice. This questionnaire measures and quantifies the nature of RMHN-SU interaction and is therefore of national interest having the potential to make more explicit and visible the skills of RMHNs, something which Brown and Fowler [21] identified as lacking as far back as 1979. The TEQ has the benefit of consisting of items and domains that are specific to a particular SU group and are therefore relevant and important to patients and clinicians [20]. Measurement tools such as the TEQ are necessary to monitor the quality of the clinical environment and help secure delivery of the best possible care to SUs.

Several practical limitations of the study should be noted. The study relied upon participants’ self-report. A substantial literature exists concerning the numerous problems of self-report data [22, 23]. Of particular concern, is the issue of social desirability which may have affected the magnitude of the results. Imminent studies of the TEQ may benefit from inclusion of other data sources like feedback via interviews with ward managers, SUs discharged and named nurses [24].

Due to the transient nature of SUs and nursing staff in these settings it was not possible to include the entire SU and nurse population eligible across all the participating Trusts. The results are only based on the ‘lived’ experience, thoughts, feelings and/or opinions of the participants recruited. Nevertheless, the study population is deemed representative of SUs and RMHNs across England so may be generalised to a large extent. More investigation is needed into the perception of nurse care delivery as they appear to view their delivery in 1-1 interactions with SUs differently to their delivery in general within the psychiatric setting as a whole.

Establishing the validity of any measurement tool is an ongoing process and future work is planned to internationally validate and implement the TEQ.

## Conclusions

The availability of a reliable and valid TE measurement tool to assess RMHN-SU interactions is central to an improved understanding of the role and contribution of RMHNs to service user recovery. We anticipate that information gathered by the TEQ will help to advise mental health nursing staff at all levels of seniority about the nature of TE experienced by SUs. We hope that the questionnaire will inform the mental health nursing profession about SU involvement in the decision-making/control over their care plan and monitoring of their treatment and/or care to ensure it is offered with dignity and compassion. The TEQ is able to determine the collaborative and empathic nature of RMHN-SU interactions, capture if SUs are treated with dignity and respect and recognise that the principles of the recovery approach are being respected.

The TEQ could help provide robust monitoring of nursing activity, offer opportunity for transparency of activity and feed into healthcare organisations' key performance indicators and other outcome measures. It will provide reassurance for Directors of Nursing about the nature and quality of nurses’ work and the degree to which they are aspiring to working in partnership with SUs as a means to enabling ‘recovery’.

## Abbreviations

RMN: Registered Mental Health Nurse
SU: Service User
